# Enhancing Forensic Diagnostics: Structured Reporting of Post-Mortem CT versus Autopsy for Laryngohyoid Complex Fractures in Strangulation

**DOI:** 10.3390/bioengineering11080807

**Published:** 2024-08-09

**Authors:** Andreas M. Bucher, Adrian Koppold, Mattias Kettner, Sarah Kölzer, Julia Dietz, Eric Frodl, Alexey Surov, Daniel Pinto dos Santos, Thomas J. Vogl, Marcel A. Verhoff, Martin Beeres, Constantin Lux, Sara Heinbuch

**Affiliations:** 1 Institute of Diagnostic and Interventional Radiology, University Hospital, Goethe University, Haus 23c, UG, Theodor-Stern-Kai 7, 60590 Frankfurt, Germanypintodossantos@med.uni-frankfurt.de (D.P.d.S.); beeres@gmx.net (M.B.); 2 Institute of Legal Medicine, University Hospital, Goethe University, Kennedyallee 104, 60596 Frankfurt, Germany; 3 Universitätsinstitut für Radiologie, Neuroradiologie und Nuklearmedizin, Johannes Wesling Universitätsklinikum Minden, Ruhr Universität Bochum, Hans-Nolte-Strasse 1, 32429 Minden, Germany; alexey.surov@muehlenkreiskliniken.de; 4 Department of Psychiatry, Clinic for Psychiatry, Psychotherapy and Psychosomatics, SHG-Kliniken Sonnenberg, Sonnenbergstraße 10, 66119 Saarbruecken, Germany

**Keywords:** post-mortem computed tomography (pmCT), autopsy, laryngohyoid complex, strangulation, fractures, structured reporting

## Abstract

Background: The purpose of this study was to establish a standardized structured workflow to compare findings from high-resolution, optimized reconstructions from post-mortem computed tomography (pmCT) with autopsy results in the detection of fractures of the laryngohyoid complex in strangulation victims. Method: Forty-two strangulation cases were selected, and pmCT scans of the laryngohyoid complex were obtained. Both pmCT scans and autopsy reports were analyzed using a structured template and compared using Cohen’s kappa coefficient (κ) and the McNemar test. The study also compared the prevalence of ossa sesamoidea and non-fusion of the major and minor horns of the hyoid bone between both diagnostic methods. Results: The detection of fractures showed a very good correlation between autopsy and pmCT results (κ = 0.905), with the McNemar test showing no statistically significant difference between the two methods. PmCT identified 28 sesamoid bones, 45 non-fusions of the major horns, and 47 non-fusions of the minor horns of the hyoid bone, compared to four, six, and zero, respectively, identified by autopsy (*p* < 0.0001). Conclusions: Autopsy and pmCT findings correlate well and can be used in a complementary manner. PmCT is superior to autopsy in identifying dislocations and detecting anatomical variations in the laryngohyoid complex, which can lead to misinterpretations during autopsy. Therefore, we do not advocate replacing autopsy with pmCT but propose using a structured workflow, including our standardized reporting template, for evaluating lesions in the laryngohyoid complex.

## 1. Introduction

The value of post-mortem computed tomography (pmCT) in forensic pathology is increasingly recognized in forensic investigations [1,2,3]. Although autopsy remains the gold standard in forensic medicine, the benefits of preceding pmCT to support and complement autopsy procedures are widely accepted [4,5,6]. Consequently, pmCT has become an integral and well-established part of the standard examination protocol in many forensic diagnostics institutions [1,2,7]. While some discussions suggest pmCT as a potential replacement for autopsy, current forensic practice predominantly adopts a complementary approach [8], underlining the growing importance and widespread use of post-mortem radiology.

Autopsy remains superior to CT in many respects, particularly in the assessment of soft tissue lesions. However, pmCT offers diagnostic advantages in several areas [8,9,10]. In particular, CT is superior in the detection of fractures and foreign materials [11,12,13]. For example, in the detection of skeletal fractures, pmCT offers higher diagnostic sensitivity compared to autopsy [8]. Imaging is preferable for accurately determining the position of foreign bodies that are at risk of positional change during autopsy, such as a bolus in the pharynx or trachea [8]. Similarly, volatile processes such as gas accumulations or fragile bone arrangements, which are challenging to assess at autopsy, are better visualized by pmCT [10]. Furthermore, the location of foreign objects, such as bullets, is more straightforward and precise with pmCT than with autopsy [9]. In addition, pmCT has been shown to be useful in identifying corpses from dental data [14] and in detecting injuries in the laryngohyoid complex [15]. In essence, pmCT complements autopsy as a non-invasive, non-destructive method that can be digitally archived, allowing for post-processing and diagnosis in a pristine state. This capability enables re-evaluation, cross-verification by other specialists, and the generation of visual evidence for use in court [3,6,9].

PmCT is particularly effective in assessing findings associated with strangulation cases. Mechanisms causing death in strangulation include airway closure, neck vein occlusion, carotid artery compression, baroreceptor stimulation (vagal inhibition), and medulla spinalis lesions [16,17]. Cartilage and osseous fractures are well visualized in pmCT. Fractures of the os hyoideum occur in approximately 30% of strangulation cases, and fractures of the larynx in more than 50% [18,19]. External signs can be minimal or even absent [3,18]. For instance, petechiae were found in the eyes in 86% of ligature strangulation cases and in 89% of manual strangulation cases during forensic examination soon after death [1,18]. Investigating ecchymosis in subcutaneous and muscle tissues, as well as hemorrhagic fractures of the hyoid bone and thyroid cartilage, is crucial during classical autopsy in strangulation cases. The detection of ecchymotic fractures in the suspended hyoid bone and thyroid cartilage and hemorrhages in the surrounding soft tissues are vital indicators of vitality [20,21]. Another rare finding in strangulation cases is lacerations in the carotid intima and hematomas in the adventitia, known as ‘amussat sign’ [22].

Signs suggestive of a strangulation incident may not be apparent in autopsy [4]. For example, if a soft instrument was used for strangulation, the typical lesions may be absent [3]. It has also been demonstrated that fractures of the anterior commissure and the cricoid cartilage are frequently unrecognized at autopsy or are overshadowed by other iatrogenic damages [3,6]. Adequate demonstration, particularly of non-dislocated fractures of the delicate structures, is prone to iatrogenic lesions due to manual examination [5]. In addition, confounding findings during autopsy may lead to misleading interpretations [23].

In radiological diagnostics, there is growing focus on the benefits of structured reporting, which is considered highly promising. Radiological societies provide resources for developing structured reporting standards and tools to enhance the value of information transfer in radiological reports [24]. The German Radiology Society has established a working group to systematize these efforts [24]. These efforts are supported by consensus meetings of specialists and the joint development of templates for reporting pancreatic lesions and cardiomyopathies [25]. While the benefits of structured reporting have been well evaluated in other diagnostic areas, this has not been extensively studied in pmCT. This feature could be particularly useful for specific evaluations, such as detecting strangulation.

Therefore, this study aimed to illustrate a standard structured workflow to compare findings from post-mortem CT and autopsy in the detection of laryngeal and hyoid fractures in a cohort of strangulation victims by comparing high-resolution, optimized reconstructions of post-mortem CTs with autopsy results of the specimens.

## 2. Materials and Methods

This monocentric retrospective study was conducted according to the Declaration of Helsinki. Approval for the retrospective analysis was obtained from the Institutional Review Board (IRB) at University Hospital Frankfurt (No. 116/14).

### 2.1. Inclusion/Exclusion Criteria

We selected a continuous cohort of strangulation cases from the database of the Institute for Diagnostic and Interventional Radiology at Goethe University (Frankfurt/Main).

The inclusion criteria specified cases with a post-mortem CT performed prior to autopsy between 1 January 2008 and 31 December 2019 and the availability of a preserved laryngohyoid complex specimen. Cases with incomplete image datasets, radiological reports, or insufficient specimens were excluded.

### 2.2. Data Generation: Imaging

The laryngohyoid complex specimens were preserved in formalin within a plastic container free of metal parts during imaging. All specimens were scanned using a Siemens Somatom Force CT scanner (Siemens, Forchheim, Germany). The positioning was aligned with the craniocaudal axis and the tracheal midline. The images were reconstructed within an 8 cm field of view. Table 1 details the acquisition and reconstruction parameters.

### 2.3. Structured Reporting: Imaging

All reports were generated using a structured template. Image datasets were analyzed by a certified and experienced radiologist with 8 years of experience in reading post-mortem CTs. The radiologist analyzed the image datasets while blinded to the autopsy reports and case identification. The anatomic regions examined were the major horns and the corpus of the hyoid bone, the superior horns and the corpus of the thyroid cartilage, as well as the cricoid cartilage. Each anatomic structure was scored according to the definitions in Table 2.

In addition, we documented the presence of ossa sesamoidea (or of so-called cartilago triticea, if not yet calcified) in the vicinity of the cornua superiores and the non-fusion (also known as articular rima [26]) of the cornua majores and minores with the corpus of the os hyoideum. The presence of these anatomic entities was documented in a binary manner (present vs. absent).

### 2.4. Data Generation: Autopsy Reports

The autopsy report was prepared in deidentified free-text format and scored according to the structured reporting template. The interpretation was performed under the direction of a forensic pathologist with 7 years of experience, who reviewed all scorings and was blinded to the evaluation of the pmCTs. To avoid ambiguity, anatomic regions for which an accurate score was not provided in the autopsy report or for which a fracture was merely suspected were excluded from scoring and the statistical analysis.

### 2.5. Study Cohort

**Figure 1:**
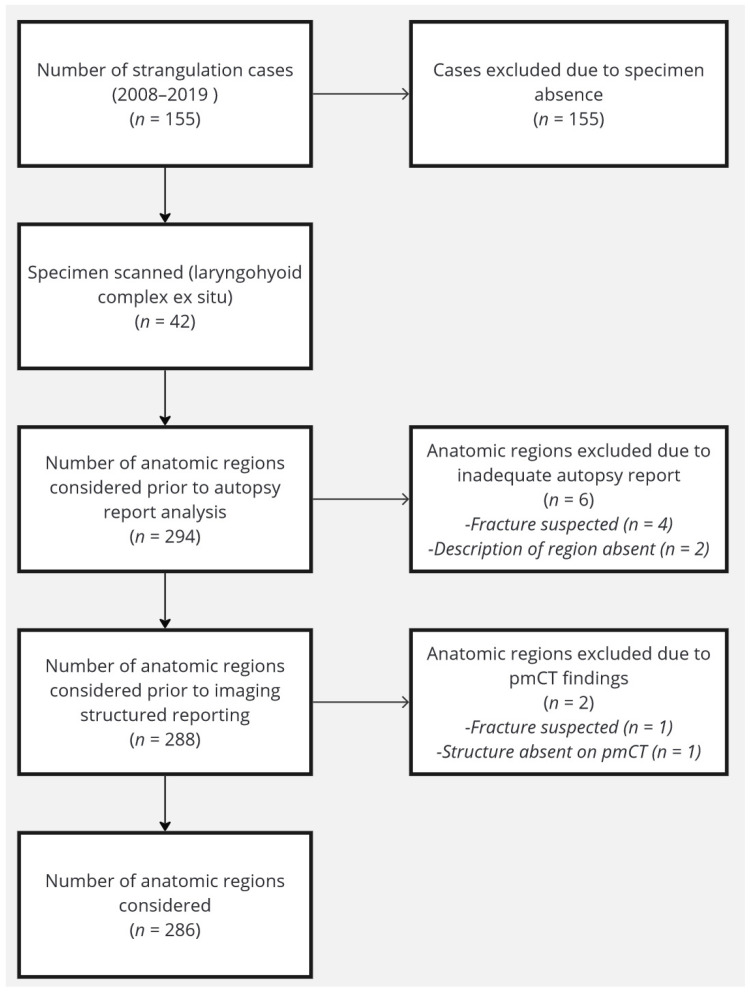
Flowchart of the study cohort.

We included 42 specimens in this analysis. For each sample, seven anatomical regions were compared (Corpus cartilago thyroidea, Cornu superius dextra, Cornus majus dextra os hyoideum, Corpus os hyoideum, Cornu majus sinistra os hyoideum, Cornu superius sinistra, and Cartilago cricoidea). Figure 1 shows the flow chart of the study. Of a total of 294 regions, 286 could be included in the analysis. Out of all regions of all datasets scanned, six anatomic samples could not be unequivocally evaluated based on the autopsy reports and were excluded from the analysis (Figure 1). Furthermore, two samples of anatomic regions had to be discarded due to the inadequate quality of their corresponding pmCT datasets (one cornua major was non-existent in the sample and in another a fracture was suspected). Overall, no pmCT dataset was discarded due to non-fulfillment of the inclusion criteria, only individual anatomic regions per CT dataset had to be excluded.

### 2.6. Statistical Analysis

Autopsy findings and pmCTs were compared using the Cohen’s kappa coefficient (κ [27]) and the NcNemar test. The kappa coefficient was evaluated using Altman’s interpretation [28]. All analyses were performed using MedCalc Statistical Software version 13.0.6 (MedCalc Software bvba, Ostend, Belgium; 2014). A *p*-value ≤ 0.05 was considered statistically significant.

## 3. Results

### 3.1. Detection of Fractures

**Figure 2:**
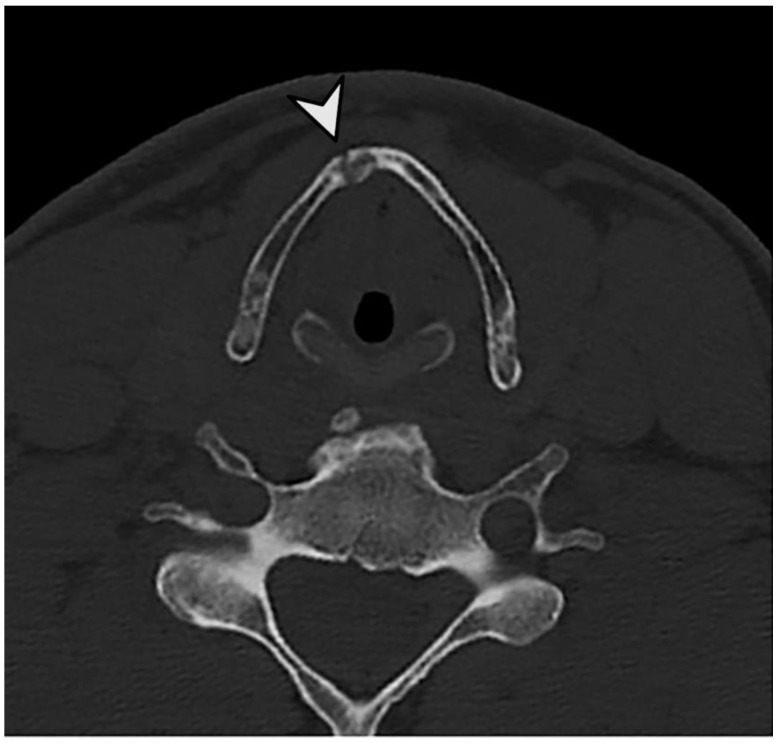
Non-dislocated fracture of the thyroid cartilage. The non-dislocated fracture is indicated by an arrowhead.

**Figure 3:**
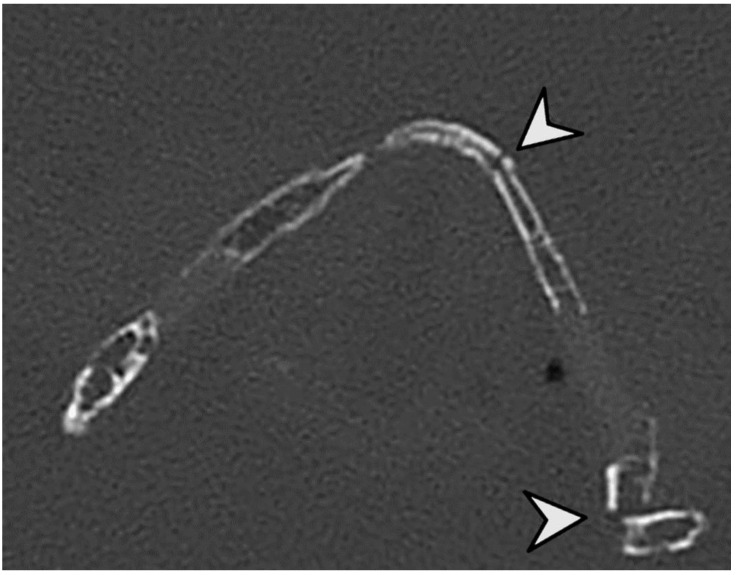
Post-mortem CT of the larynx after reconstruction, where two fractures are visible (in the corpus and the cornu superius). The two fractures are indicated by arrowheads.

**Figure 4:**
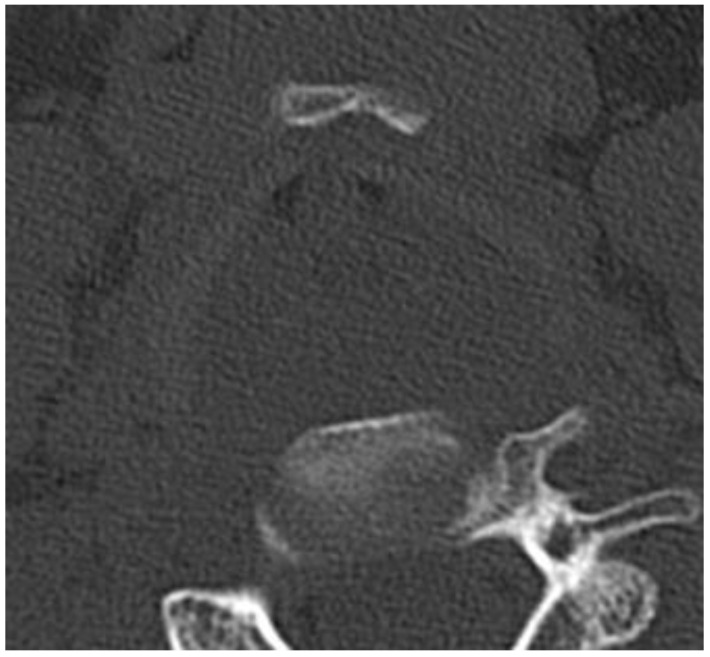
Axial CT section on the height of the thyroid cartilage. The cartilage in this sample is not calcified. Non-calcified structures posed a limitation in our study, since non-displaced fractures in these samples are not readily visualized.

**Figure 5:**
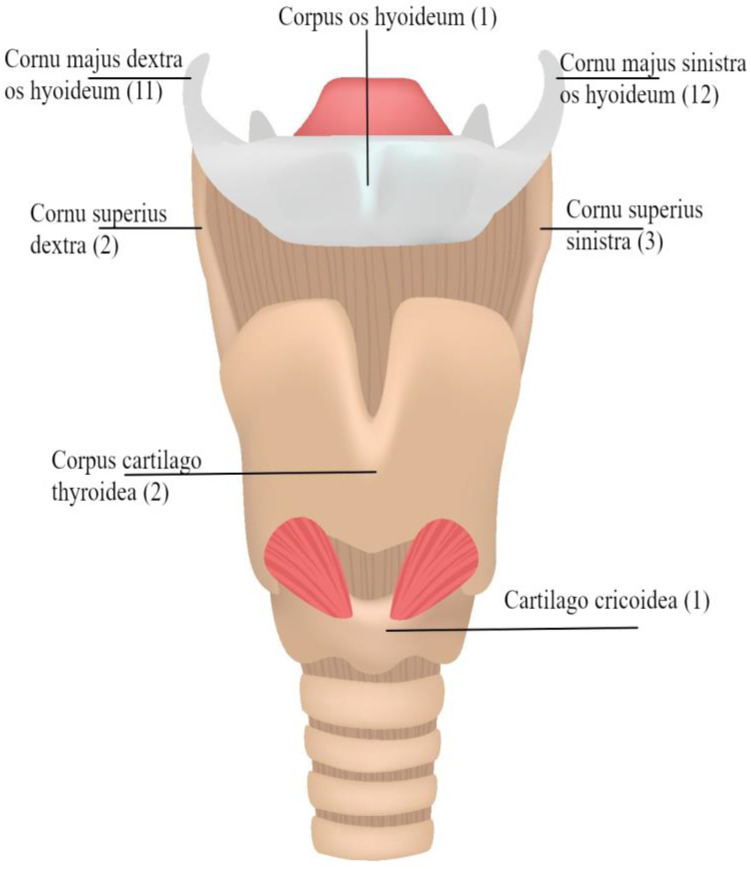
Anatomic regions compared via autopsy and pmCT post-mortem. The number of fractures identified on pmCT is shown in parentheses. Non-OA publishing license: Adapted with permission from Shutterstock, Copyright 2017 (Standard License), Copyright Owner’s Name: Achiichiii, https://www.shutterstock.com/license, accessed on 25 June 2024.

In this study, we investigated 42 specimens, which resulted in a total of 286 evaluable anatomical regions. The seven anatomical regions Cornu majus dextra, Conus majus sinistra, Cornu superius dextra, Cornu superius sinistra, Corpus os hyoideum, Corpus cartilago thyroidea and Cartilago cricoidea were analyzed. The analysis revealed that post-mortem CT and autopsy both demonstrated a high degree of accuracy in the detection of fractures within the laryngohyoid complex (Figure 2, Figure 3 and Figure 4). Specifically, 37 fractures were detected by autopsy (12.9%), while pmCT identified 35 fractures (12.2%). The distribution of fractures across different anatomical regions is shown in Figure 5, with the majority being located in the cornua superiores of the thyroid cartilage. This pattern of distribution indicates a higher prevalence of fractures in the superior structures, with a decreasing frequency in the more caudally located cartilaginous structures and the hyoid bone (Table 3).

Statistical analysis using Cohen’s kappa coefficient demonstrated excellent agreement between pmCT and autopsy findings (κ = 0.905), indicating strong consistency between both methods. In addition, the McNemar test indicated no statistically significant difference between the two diagnostic methods, with a discrepancy of only 0.70% (Table 4). This high level of concordance underscores the reliability of pmCT as a complementary tool to traditional autopsy in identifying fractures.

### 3.2. Detection of Normal Variants

The study also explored the prevalence of normal anatomical variants detectable by pmCT, which are potential confounding factors during autopsy. pmCT identified ossa sesamoidea in 42.9% of cases (unilateral: 19%; bilateral: 23.8%), non-fusion of the cornua majores in 61.9% (unilateral: 16.7%; bilateral: 45.2%), and non-fusion of the cornua minores in 73.7% (unilateral: 23.7%; bilateral: 50%) of cases (Table 5).

In contrast, the autopsy described only four sesamoid bones, six non-fusions of the major horns, and no non-fusions of the minor horns. The discrepancies between pmCT and autopsy were statistically significant, as evidenced by the McNemar test (*p* < 0.0001), with differences of 28.57% for sesamoid bones, 42.1% for non-fusions of the major horns, and 64.4% for non-fusions of the minor horns (Table 6). Cohen’s kappa coefficient for these findings was significantly lower (κ = 0.173 for sesamoid bones, κ = 0.158 for major horns, and κ = 0.0 for minor horns), indicating less agreement between the two methods in detecting these variants.

### 3.3. Evaluation of Specific Cases

Among the two hundred eighty-six evaluated regions, six regions could not be unequivocally scored based on autopsy reports and were excluded from the analysis. These exclusions primarily stemmed from ambiguities in the free-text autopsy reports where fractures were suspected but not definitively described. Instead, terms such as hypermobility of the hyoid bone were used, which did not correlate with clear anatomical descriptions. It is notable that pmCT identified relevant anatomical entities in all of these cases, which were recorded as non-fusion in the final analysis.

**Figure 6:**
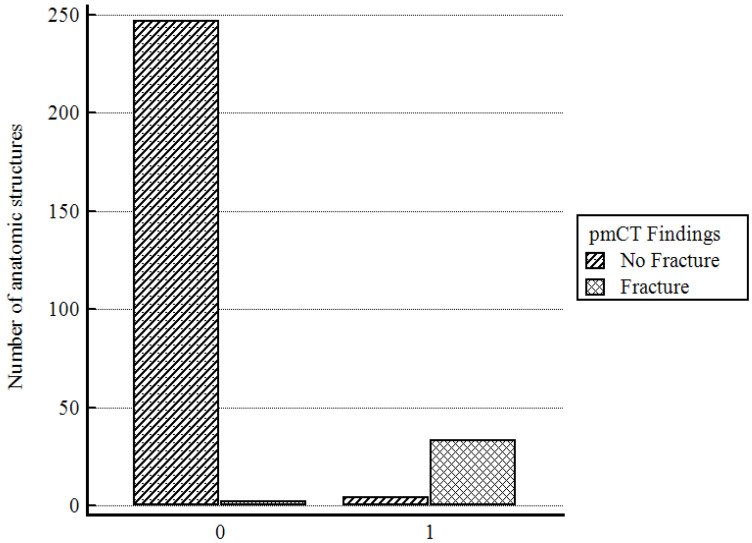
Accuracy in detection of fractures, showing (from left to right) true negative (*n* = 247), false positive (*n* = 2), false negative (*n* = 4), and true positive (*n* = 33) findings for both CT and autopsy. This representation assumes autopsy findings to be the gold standard; the number of true negatives is represented by the bar on the far left and true positives on the far right. There were very few false positives (*n* = 2) and false negatives (*n* = 4). (The X-axis represents the findings from the autopsy: 0 = No Fracture, 1 = Fracture).

Figure 6 illustrates the accuracy in fracture detection, presenting true negative, false positive, false negative, and true positive findings for both pmCT and autopsy. The results indicate a low number of false positives (*n* = 2) and false negatives (*n* = 4), reinforcing the diagnostic accuracy of pmCT in detecting fractures in comparison to autopsy.

### 3.4. Detailed Findings

Table 3 presents a breakdown of the fracture frequencies across different anatomical regions. Most fractures were located in the cornua superiores of the thyroid cartilage. The high prevalence of fractures in this region highlights its vulnerability during incidents of strangulation. Additionally, the corpus cartilago thyroidea, cartilago cricoidea, and os hyoideum exhibited notable, albeit lower, frequencies of fractures. These findings are in accordance with the anatomical distribution of stress during strangulation.

Table 4 provides a statistical comparison between pmCT and autopsy fracture findings, indicating a high level of agreement and no significant discrepancies between pmCT in forensic examinations and autopsy reports.

Table 5 summarizes the prevalence of anatomical variants detected by pmCT that could potentially confound autopsy results. There were significant differences in detection rates between pmCT and autopsy, demonstrating that pmCT has a higher sensitivity in identifying these variants.

Table 6 presents a statistical comparison of normal variant findings between pmCT and autopsy, for which the detection capability of pmCT was higher compared to routine autopsy reports.

## 4. Discussion

PmCT has become a standard method in forensic reconstructive workups. In light of this, we compared the accuracy in identifying fractures of the laryngohyoid complex in strangulation cases using pmCT imaging applied to autopsy findings. While we observed a good correlation between both methods, we also identified deviations. We demonstrated the clear superiority of pmCT in detecting anatomic variants. Therefore, we suggest a complementary approach that uses a structured reporting template to guide autopsy in all suspected strangulation cases.

Studies that have compared the accuracy of pmCT and autopsy in detecting fractures in the laryngohyoid complex in strangulation incidents have generally found a good correlation between the two methods. Deininger et al. reported, among other findings, that there was an agreement in the diagnosis of fractures between pmCT and autopsy in 72–80% of cases, depending on the examined anatomic region [29]. Blanc-Louvry et al. found a good correlation between pmCT and autopsy findings for fractures of the hyoid bone [30]; Yen et al. [31] reported similar findings for fractures in the laryngohyoid complex. Treitl et al. reported a kappa coefficient of 0.762 between pmCT and autopsy pertaining to fracture diagnosis, compared to 0.905 in our study [15]. A meta-analysis on the concordance of pmCT and autopsy found both methods to be comparable regarding fracture identification [32].

Our study revealed discrepancies in six cases. The two fractures identified on pmCT, which were not described during autopsy, were discussed in detail with the forensic pathologist. These could be attributed to iatrogenic damage resulting from the dissection technique used to remove the tissue en bloc prior to pmCT. After exclusion of these cases from the statistical analysis, there were no false positive findings on pmCT. Conversely, four fractures described in the autopsy report were not identified in pmCT. In three cases, the thyroid cartilage was not sufficiently calcified and, therefore, could not be adequately scored by means of the pmCT available (Figure 3). This limitation is in line with the findings of Decker et al., who concluded that imaging was superior to autopsy in the diagnostics of calcified tissues but less accurate in the detection of other lesions [2]. The remaining case pertained to a major horn of the thyroid cartilage, where a sesamoid bone was also present.

Knowledge of the presence of anatomic deviations is important in forensic medicine [23]. A radiologic report prior to autopsy provides the pathologist with valuable information in this regard. Triticeal cartilage in the vicinity of the superior horns of the thyroid cartilage may be mistaken for a fracture, particularly if its calcification is advanced substantially [23]. With regard to the detection of anatomic deviations by autopsy, as described in Table 6, one reason for the discrepancy is that in our department of forensic medicine, a sesamoid bone, though easily detectable, is often excluded from the autopsy report, mainly for the sake of relevance and brevity. A number of studies have examined the prevalence of sesamoid bones/triticeal cartilages, using various methods, resulting in a wide range of findings, ranging from 8.1 to 65% [15,23,26,33,34,35]. Overall, the results of our study (prevalence 42.9%) are consistent with the majority of the studies conducted in European cohorts [15,23,26,33]. Ethnicity is assumed to be a factor in their prevalence [33].

Di Nunno et al. [26] identified 35% of non-fusion of the greater horn of the hyoid bone to the body and 60% of non-fusion of the minor horns. We found the prevalence of the greater horn to be higher (61.9% of specimens, Table 5), while the prevalence of the minor horn (73.7%) was is in accordance with these findings. An anatomic variation in the laryngohyoid complex was identified by de Bakker et al. in 63% of samples, while clinically relevant variations that can introduce a confounding factor in the diagnosis of potential fractures were found in 19% [23], but they did not examine possible non-fusions of the major and minor horns of the hyoid bone. Fusion varies according to age and gender. It is, therefore, recommended that PmCT cases should be analyzed in a more differentiated manner [36].

One contributing factor for the discrepancies between autopsy and pmCT results is iatrogenic damage, which we also found in our study. This possibility is also discussed in the literature [3,32]. Manual testing during autopsy has the risk of damaging and breaking delicate anatomic structures. This is particularly likely to occur when specimens are taken. This risk is non-existent during tomographic examination, due to its non-invasive nature [3].

Despite these factors, discrepancies were found in only five of forty-two cases (11.9%) in our sample. In 88.1% of the cases, there was a complete agreement of findings between pmCT and autopsy, which is higher than the 75% reported by Kempter et al. [3].

## 5. Limitations

We acknowledge several limitations in our study design. Due to the retrospective nature of this study, only a limited number of preserved specimens were available for re-examination using new high-resolution pmCT. Although an experienced forensic pathologist assisted in the evaluation, there were cases where the text could not be unambiguously scored according to the scheme used for pmCTs. While a structured reporting template requires all regions to be scored, even if they appear “normal”, a free-text report may omit such details, which could lead to ambiguities when read later. Despite this limitation, the vast majority of all free-text autopsy reports were fully compatible with the template used, reflecting the high standard of free-text reporting at autopsy. This issue could have been mitigated if a common template for reporting findings had been used across the university clinic. The structured reporting according to pmCT is based on visual facts that can be directly scored, whereas free-text autopsy reports can be interpreted in different ways. Although these documentation sources are inherently different, they represent real-life workflows. It is crucial to determine the accuracy and correlation of these two methods in advance, as court cases may rely on both autopsy reports and pmCT data.

## 6. Future Directions

Future research should aim to include larger sample sizes in order to enhance the statistical power and generalizability of findings. Furthermore, additional studies are essential to refine and validate structured reporting templates for pmCT, in line with those used in other areas of radiology and forensic medicine. These templates should be standardized and widely adopted in order to ensure consistency and accuracy in forensic diagnostics.

Investigating the potential of advanced imaging techniques, such as dual-energy CT or MRI, in combination with pmCT could provide deeper insights into soft tissue and bone pathology, potentially overcoming some of the limitations identified in the current study. Furthermore, collaborative studies involving multiple institutions could also help to establish universal guidelines and protocols, facilitating better integration of pmCT into routine forensic practice.

### Recommendations for Routine Workflows in Forensic Imaging

1. Complementary Use of Autopsy and pmCT: Both autopsy and pmCT provide valuable and complementary insights into the detection of fractures and anatomical variations in the laryngohyoid complex. The high correlation between the two methods supports their combined use in a comprehensive forensic evaluation.
2. Superiority of pmCT in Detecting Anatomic Variations: pmCT has demonstrated superior sensitivity in identifying anatomical variations and non-fusions that can be confounding factors during autopsy. It is particularly effective in the detection of fracture dislocations and minor anatomical deviations that may be overlooked during manual examination.
3. Standardized Reporting Template: Implementing a standardized reporting template is recommended for pmCT examinations of the laryngohyoid complex. This template should be used to document all relevant anatomical regions in a systematic manner, ensuring thorough examination and facilitating clear communication between radiologists and forensic pathologists.
4. Pre-Autopsy pmCT Examination: Conducting pmCT examinations prior to autopsy is strongly recommended in suspected cases of strangulation. The non-invasive nature of pmCT allows for the preservation of the anatomical state, enabling detailed analysis and providing critical information that can guide the autopsy process.

Integrating these recommendations into forensic practice will significantly improve the diagnostic accuracy and reliability of forensic examinations, ultimately contributing to more precise and informed legal and medical outcomes.

## 7. Conclusions

The pmCT diagnostics correlated well with autopsy findings and can provide important additional information to the forensic pathologist. It is, therefore, strongly recommended that such an examination be performed prior to autopsy in cases where strangulation is suspected. 

## Figures and Tables

**Table 1:** PmCT acquisition and reconstruction parameters.

Parameter	Value
Slice thickness	1 mm
Increment	0.8 mm
Orientation	Z-axis parallel to tracheal axis
Sections	axial, coronal, sagittal
Kernel	Br69, H40

**Table 2:** Excerpt of the coding system used in the study. A score from 0 to 2 was awarded for each anatomical structure.

Score	Interpretation
0	No Fracture
1	Fracture present
2	Fracture suspected but not clearly visible on the pmCT or not unambiguously described on the autopsy report

**Table 3:** Frequencies of detected fractures in each of the regions studied. The regions represented here correspond to the seven regions defined for structured reports of pmCT, these are illustrated in Figure 5.

	Autopsy *n* (%)			pmCT *n* (%)		
	Right	Middle	Left	Right	Middle	Left
Cornua superiores	10 (23.8%)	-	12 (28.6%)	10 (23.8%)	-	10 (23.8%)
Corpus cartilago thyroidea	-	6 (14.3%)	-	-	5 (11.9%)	-
Cartilago cricoidea	-	1 (2.4%)	-	-	2 (4.8%)	-
Os hyoideum	4 (9.5%)	1 (2.4%)	3 (7.1%)	4 (9.5%)	1 (2.4%)	3 (7.1%)

**Table 4:** Statistical comparison between pmCT and autopsy fracture findings.

	Result	SE	95% CI	*p*
Cohen’s κ	0.905	0.04384	0.829 to 0.980	N/A
McNemar	0.70%	N/A	−1.16 to 1.92	0.6875

**Table 5:** Prevalence of the examined anatomic entities in pmCT that can potentially be a confounding factor during autopsy.

	Right	Left	Bilateral Localization in Tissue Sample	Unilateral Localization in Tissue Sample	Tissue Samples with Anatomic Variants
Os Sesamoideum	35.7% (*n* = 15/42)	31% (*n* = 13/42)	23.8% (*n* = 10/42)	19% (*n* = 8/42)	42.9% (*n* = 18/42)
Non-fusion cornua majores	52.4% (*n* = 22/42)	54.8% (*n* = 23/42)	45.2% (*n* = 19/42)	16.7% (*n* = 7/42)	61.9% (*n* = 26/42)
Non-fusion cornua minores	59.5% (*n* = 22/37)	65.7% (*n* = 25/38)	50% (*n* = 19/38)	23.7% (*n* = 9/38)	73.7% (*n* = 28/38 ^1^)

^1^ A total of 5 right and 4 left cornua minores were missing and, thus, excluded from the count.

**Table 6:** Statistical comparison between pmCT and autopsy normal variant findings. The percentage refers to the total number of structures examined (normally 2 per tissue sample, if both structures are present during examination).

	Count (pmCT)	Count (Autopsy)	Correlation (κ)	Correlation (McNemar)
Os Sesamoideum	33.3% (*n* = 28/84)	4.8% (*n* = 4/84)	0.173 (95% CI: 20.43 to 28.57)	28.57% (*p* < 0.0001)
Non-fusion cornua majores	53.6% (*n* = 45/84)	7.1% (*n* = 6/84)	0.158 (95% CI: 0.0382 to 0.278)	42.1% (*p* < 0.0001)
Non-fusion cornua minores	62.7% (*n* = 47/75)	0.0% (*n* = 0/75)	0.0 (95% CI: 0.0 to 0.0)	64.4% (*p* < 0.0001)

## Data Availability

The datasets presented in this article are not readily available because there are data protection issues and CT images cannot be assumed to be anonymized datasets.
